# ‘I know it’s not normal but it’s normal to me, and that’s all that matters’: experiences of young adults conceived through egg donation, sperm donation, and surrogacy

**DOI:** 10.1093/humrep/dead048

**Published:** 2023-03-15

**Authors:** V Jadva, C Jones, P Hall, S Imrie, S Golombok

**Affiliations:** Centre for Family Research, University of Cambridge, Cambridge, UK; Institute for Women’s Health, University College London, London, UK; Institute for Women’s Health, Faculty of Population Health Sciences, University College London, London, UK; Social, Genetic & Developmental Psychiatry Centre, King’s College London, London, UK; Centre for Family Research, University of Cambridge, Cambridge, UK; Centre for Family Research, University of Cambridge, Cambridge, UK; Murray Edwards College, University of Cambridge, Cambridge, UK; Centre for Family Research, University of Cambridge, Cambridge, UK

**Keywords:** surrogacy, egg donation, sperm donation, young adults, experiences, third-party

## Abstract

**STUDY QUESTION:**

What are thoughts and feelings of young adults born following egg donation, sperm donation, and surrogacy?

**SUMMARY ANSWER:**

Young adults felt either unconcerned or positive about the method of their conception.

**WHAT IS KNOWN ALREADY:**

Much of what we know about adults born to heterosexual couples following anonymous donation has come from samples of donor conceived people who had found out about their origins during adulthood. There have been no studies of how young adults born through surrogacy feel about their conception and towards their surrogate.

**STUDY DESIGN, SIZE, DURATION:**

Thirty-five young adults were interviewed as part of the seventh phase of a larger multi-method, multi-informant longitudinal study of assisted conception families in the UK. Adults were conceived using either egg donation, sperm donation, gestational surrogacy, or genetic surrogacy and were raised in households headed by heterosexual couples.

**PARTICIPANTS/MATERIALS, SETTING, METHODS:**

Participants had a mean age of 20 years and were born following traditional surrogacy (n = 10), gestational surrogacy (n = 5), egg donation (n = 11), or sperm donation (n = 9). All young adults born following sperm donation and most (n = 10) born from egg donation had an anonymous donor. In all surrogacy arrangements, the parents had met the surrogate prior to treatment. The majority of young adults were told about their conception by the age of 4 years. Participants were interviewed over the internet using a semi-structured interview. Interviews were transcribed verbatim and analysed using qualitative content analysis to understand young adults’ thoughts and experiences related to their conception and whether they were interested in meeting their donor or surrogate.

**MAIN RESULTS AND THE ROLE OF CHANCE:**

Fourteen (40%) young adults felt their conception made them feel special or unique, with the remainder feeling either neutral or unconcerned (n = 21, 60%). A higher proportion of young adults conceived using egg donation (n = 8, 73%) felt unique/special compared to young adults born following sperm donation and surrogacy. For 10 of the young adults, their feelings about their conception had changed over time, with most becoming more positive (n = 9, 26%). For most young adults (n = 22, 63%), conception was rarely or infrequently discussed with others. However, when it was, these conversations were largely conducted with ease. Most (n = 25, 71%) did not know other individuals born through the same method of conception as themselves, and the vast majority (n = 34, 97%) were not members of any support groups. For the 25 young adults not in contact with their donor or surrogate, 11 wished to meet them, 8 did not want to have contact, and 6 were unsure. Young adults in contact with their donor or surrogate had varying levels of closeness to them. Only one young adult had searched for the identity of their donor.

**LIMITATIONS, REASONS FOR CAUTION:**

Of the 47 young adults invited to participate in the present study, 35 agreed to take part resulting in a response rate of 74%. It is therefore not known how those who did not take part felt about their conception. Given that the families reported here had been taking part in this longitudinal study from when the target child was aged 1 year, they may have been more likely to discuss the child’s conception than other families. The study also utilized self-report measures, which may have been prone to social desirability, with donor conceived young adults wanting to present their experiences in a positive light.

**WIDER IMPLICATIONS OF THE FINDINGS:**

The findings suggest that young adults born through surrogacy and donor conception do not feel negatively about their birth and this may be a consequence of the young age at which they found out about their conception. Although some young adults said they wished to meet their donor, this did not necessarily mean they were actively searching for them.

**STUDY FUNDING/COMPETING INTEREST(S):**

The study was funded by the Wellcome Trust [grant number 208013/Z/17/Z]. The authors have no conflicts of interest to declare.

**TRIAL REGISTRATION NUMBER:**

N/A.

## Introduction

Despite the concerns raised about how children born following third-party reproduction would feel about their conception, empirical research in this area remains limited. For individuals born following anonymous egg and sperm donation, concerns have included how they would feel about lacking a genetic connection with one of their parents and not knowing the identity of their donor (Golombok, 2021). For individuals born following surrogacy, questions remain about whether they feel distressed in adulthood by the knowledge that their surrogate gave them away to their intended parents and, for those with a known donor or a surrogate, whether this relationship is maintained over time and, if so, how the young person feels about ongoing contact (Brazier *et al.*, 1998).

Studying the perspectives of individuals born following donor conception and surrogacy has been inherently difficult owing to the high rates of non-disclosure of the child’s origin in families headed by heterosexual couples (Lassalzede *et al.*, 2017). Compared to offspring in families headed by single women and same-sex couples, individuals conceived by gamete donation to heterosexual couples who have been told, report finding out about their conception at a later age (Jadva *et al.*, 2009). Families formed through surrogacy show higher rates of disclosure, perhaps because surrogacy is more difficult to conceal from others as the parents must explain the arrival of a baby without having experienced a pregnancy (MacCallum *et al.*, 2003). Non-disclosure runs the risk of the child finding out accidently or at a later age, which has been associated with more negative feelings about donor conception, including feelings of anger, shock, and confusion (Turner and Coyle, 2000; Jadva *et al.*, 2009; Beeson *et al.*, 2011). In contrast, finding out in the preschool years has been found to be associated with more positive mother–child relationships in adolescence (Ilioi *et al.*, 2017), and early adulthood (Golombok *et al.*, in press), and can enable the information to be incorporated into the child’s sense of identity (Rumball and Adair, 1999). Some donor conceived individuals born following anonymous or identity-release donation have been found to express curiosity about their conception (Jadva *et al.*, 2009, Scheib *et al.*, 2005; Rodino *et al.*, 2011), which, for some, leads to seeking information about their donor and/or their donor siblings (Macmillan *et al.*, 2021). It is important to note that most studies of donor conceived adults conceived using anonymous donation have used volunteer samples, sometimes of people who have joined support groups for donor-conceived people, and thus their experiences of donor conception may be different from those of donor conceived people who do not join these groups.

The UK Longitudinal Study of Assisted Reproduction Families has followed families born through egg donation, sperm donation and surrogacy from infancy to adulthood (Golombok *et al.*, in press; Golombok, 2021). The families have been seen at seven time points when the children were aged 1, 2, 3, 7, 10, 14, and 20 years. In relation to how the children felt about their conception, findings from age 7 years showed that children born through egg and sperm donation understood very little (Blake *et al.*, 2010). By age 10 years, some children used terms such as eggs or seed to explain their conception, but not all children were able to explain their conception to the researcher (Blake *et al.*, 2014). In contrast, the vast majority of children born through surrogacy (90% n = 19) had a good understanding of their conception at ages 7 and 10 years, explaining that they were born to someone other than their mother (Jadva *et al.*, 2012). At age 14 years, the majority of adolescents interviewed felt unconcerned or positive about their conception, and those not in contact with their surrogate or donor were interested in them. For example, some wanted to know their motivations for being a donor or surrogate. Of the 16 adolescents who were in contact with their surrogate or donor, 14 expressed positive feelings towards them, 1 felt ambivalent, and 1 felt negative following a breakdown in their relationship (Zadeh *et al.*, 2018). Although most appeared to feel either positive or unconcerned about the use of third-party reproduction, it is important to note that feelings about conception may change as children become adults and form their own independent narratives about their birth, which may differ from that explained to them by their parents (Jadva *et al.*, 2012).

The present study reports on data from the 7th phase of the UK Longitudinal Study of Assisted Conception Families when the target child was a young adult. Emerging adulthood (the age period from late teens to mid-20’s) is an important developmental phase; issues around identity formation remain important as young adults continue to develop autonomy, progress towards self-sufficiency, and understand themselves and others better (Arnett, 2004, 2007). For donor conceived and surrogacy born young adults, this developmental period can be important as they may begin to have more independent thoughts about their donor or surrogate and, for those not in contact with them, start to think autonomously about whether they wish to seek information and/or contact them.

To our knowledge, this is the first study of young adults born through surrogacy. In the absence of empirical research on how young adults feel about their conception following surrogacy, the present article aims to provide insight into their thoughts and feelings about their birth, and towards their parents and surrogate. Furthermore, only a handful of studies have examined the thoughts and experiences of donor conceived individuals who have been aware of their conception from a young age. Given the recent move towards greater openness, and the increasing numbers of parents telling their children about their conception (Tallandini *et al.*, 2016), understanding the views of these young adults is important to better support parents and their children. Furthermore, the law in the UK on donor anonymity changed in 2005 and all clinic donors are now identity release donors. This means that a donor conceived individual conceived after 1 April 2005 has the right to request identifying information about their donor at age 18 years. In addition, currently in the UK, the Human Fertilisation and Embryology Authority (HFEA) is questioning whether donor anonymity should be removed ‘at use’, i.e. at the point of treatment, rather than waiting until the age of 18 years (Chain, 2022). These discussions are linked to wider debates about whether donors can be anonymous given the ease of direct-to-consumer DNA testing (Harper *et al.*, 2016; Newton *et al.*, 2022). Understanding how these young adults feel about their conception, and whether they wish to seek information about their donor and, indeed, wish to meet them, is therefore of paramount importance for informing these debates.

## Material and methods

### Sample characteristics

Data were drawn from the seventh phase of the UK Longitudinal Study of Assisted Reproduction Families (Golombok *et al.*, in press; Golombok, 2021). Fifty sperm donation families, 51 egg donation families, 42 surrogacy families, and 80 natural conception families, all headed by heterosexual couples, were first visited when the target child was aged 1 year (Golombok *et al.*, 2004a,b) and data were subsequently collected at age 2 years (Golombok *et al.*, 2005, 2006a), 3 years (Golombok *et al.*, 2006b), 7 years (Golombok *et al.*, 2011), 10 years (Golombok *et al.*, 2013), 14 years (Golombok *et al.*, 2017), and 20 years (Golombok *et al.*, in press). With the exception of a small number of egg donation and surrogacy families who had used known egg donors, the families had used clinic donors prior to the 2005 change in the law and had thus used anonymous donors. The surrogacy families in the study had used either traditional surrogacy using the surrogate’s egg, or gestational surrogacy using the egg and sperm of the intended parents.

The gamete donation families were recruited through fertility clinics, and the surrogacy families were recruited through the Office for National Statistics, which, at the time, kept a record of all applications for a Parental Order—the legal process for transferring parentage from the surrogate to intended parents. Additional surrogacy families were recruited through Childlessness Overcome Through Surrogacy—the only surrogacy support group in existence at that time. Details of the original recruitment process can be found in Golombok *et al.* (2004a,b). The present article reports on data from the seventh phase of this larger longitudinal study when the target child had reached adulthood.

Of the 47 young adults who had been informed about their method of conception, 35 agreed to take part in the study giving a response rate of 74%. Forty-four participants had taken part in the previous phase of the study when the children were aged 14 years (Zadeh *et al.*, 2018). The present sample comprised 15 young adults born through surrogacy, 11 through egg donation, and 9 through sperm donation. All young adults born following sperm donation, and most (n = 10) born from egg donation, had an anonymous donor. Five of the participants had been conceived using gestational surrogacy (using the intending parents’ gametes) and 10 using traditional surrogacy (where the surrogate’s egg was used for the pregnancy). In all surrogacy arrangements, the parents had met the surrogate prior to treatment. Most young adults had been informed about their conception during childhood, usually by age 4 years (Ilioi *et al.*, 2017). Demographic details of participants can be seen in Table I. There was an almost even split of participants by gender, with 49% (17) male and 51% (18) female participants. The large majority of the sample identified their ethnicity as White, with a minority of participants not stating their ethnicity, and one stating that half of their ethnicity was unknown. The largest proportion of participants was studying full-time (46%, n = 16).

**Table I:** Characteristics of participants in a study of experiences of young adults conceived through egg donation, sperm donation, and surrogacy.

	Sperm donation	Egg donation	Surrogacy	Total
Age (years, mean, SD)	19.38 (0.74)	19.73 (0.65)	19.87 (0.83)	19.71 (0.76)
Sex				
Male	5	6	6	17
Female	4	5	9	18
Ethnicity				
White	7	10	10	27
Other	0	0	2	2
Unknown	1	0	0	1
Missing	0	1	3	4
Working status				
Working	1	1	4	6
Full-time student	5	3	8	16
Working and studying part-time	1	6	2	9
Other	2	1	1	4

All data, except age, are *n* values.

The present article reports on data obtained during the interviews with the young adults. owing to the coronavirus disease 2019 pandemic, all interviews were conducted over Zoom (a videotelephony software program: Zoom Video Communications, San Jose, CA, USA) by two of the authors (C.J. and P.H.) who are trained research psychologists. As the interviewers were themselves young adults, they had an insider status regarding the participants’ identities, which helped build rapport with the interviewees. A section of the interview asked young adults about their thoughts and experiences related to their conception, including how they felt about their conception, whether they discussed this with anyone, and how others reacted. They were also asked whether they were interested in meeting their donor or surrogate or, if already in contact, about their relationship and feelings towards them. Finally, they were asked whether they knew others born using the same method of conception as themselves, and if they were a member of any support groups. This section of the interview was transcribed verbatim. Ethical approval for this study was obtained from the University of Cambridge Psychology Research Ethics Committee. During the consent process, participants were informed that the interviews would be transcribed and that some data may be reported in our publications.

### Analysis plan

Interviews were analysed using both inductive and deductive qualitative content analysis (Krippendorff, 2004). This approach reflects the researchers’ critical realist position and their commitment to participants’ interpretations of their experiences. Each transcript was carefully read by the first author and text-driven codes were produced to capture the content of the transcripts. The interviews were then rated according to these codes and frequency counts calculated. The software package ATLAS.ti 9 Scientific Software Development GmbH, Berlin, Germany was used to aid analysis. The following ratings were made: Current feelings about conception (Unconcerned, Feeling special/unique); Change in feelings over-time (Yes—More positive, Yes—More negative, No); Frequency of discussions (Frequently, Fairly Often, Infrequently, Very rarely); Ease of discussions (Difficult, Mixed, Easy, Really Easy); Openness of communication (Mother, Father, Parents, Siblings, Close-friends, Anyone); Whether lack of genetic/gestational connection affected relationship to mother/father (Yes, No); Know others conceived in same way (Yes, No); Whether others understand conception method (Yes, No); Member of donor conception or surrogacy group (Yes—in the past, No); Searched for information on conception method (Yes, No); and Contact with donor or surrogate (Yes/No). The codes were checked by a second coder to ensure the categories accurately reflected the content of the interviews. Illustrative codes are reported in the Results section.

## Results

### Current feelings about conception

Most participants, especially those born using surrogacy, expressed a lack of concern about the method of their conception (Table II). This was usually explained by using words such as ‘not caring’, or ‘It doesn’t really bother me’. For example, one young adult said:

*‘It doesn’t faze me really, people are born in all different ways and if I was born a little bit differently - that’s OK, I understand’.* Young adult born though gestational surrogacy

Similarly, another young adult born through sperm donation reported:

*‘I've never really thought about it in a way that, that like, my dad’s my dad, my mum’s my mum, I've never really thought about how anything’s different so, it's hard to put, I don’t really care’.* Young adult conceived using sperm donation

Young adults born following egg donation were more likely (73%, n = 8) to report that their method of conception made them feel unique or special. For example, one young adult born through egg donation said:

*‘I think it was amazing, I think the whole thing is absolutely incredible. Erm … I don’t have anything negative to say about it at all’.*

**Table II:** Feelings about conception, telling others, impact on relationship with parents, and searching for information.

	Sperm donation	Egg donation	Gestational surrogacy	Traditional surrogacy	Total
	N (%)	N (%)	N (%)	N (%)	N (%)
Feelings about conception					
Unconcerned	5 (56)	3 (27)	4 (80)	9 (90)	21 (60)
Feeling special/unique	4 (44)	8 (73)	1 (20)	1 (10)	14 (40)
Change in feelings about conception over time					
Yes—more positive	2 (22)	4 (36)	0 (0)	3 (30)	9 (26)
Yes—more negative	1 (11)	0 (0)	0 (0)	0 (0)	1 (3)
No	6 (67)	7 (64)	5 (0)	7 (70)	25 (71)
Frequency of discussions					
Frequently	2 (22)	1 (9)	0 (0)	0 (0)	3 (9)
Fairly often	2 (22)	3 (27)	1 (20)	5 (50)	11 (31)
Infrequently	3 (33)	5 (45)	1 (20)	5 (50)	15 (43)
Very rarely	2 (22)	2 (18)	3 (60)	0 (0)	7 (20)
Ease of discussions					
Awkward	1 (11)	0 (0)	0 (0)	0 (0)	1 (3)
Mixed	0 (0)	3 (27)	0 (0)	3 (30)	6 (17)
Easy	4 (44)	4 (36)	3 (60)	5 (50)	16 (46)
Really easy	4 (44)	4 (36)	2 (40)	2 (20)	12 (34)
Openness of communication					
Mother	5 (56)	3 (27)	2 (40)	4 (40)	14 (40)
Father	0 (0)	0 (0)	0 (0)	0 (0)	0 (0)
Parents	1 (11)	2 (18)	1 (20)	2 (20)	6 (17)
Siblings	1 (11)	2 (18)	1 (20)	2 (20)	6 (17)
Friends	0 (0)	2 (18)	0 (0)	0 (0)	2 (6)
Close friends & family	1 (11)	1 (9)	1 (20)	1 (10)	4 (11)
Anyone	1 (11)	1 (9)	0 (0)	1 (10)	3 (9)
Whether lack of genetic/gestational connection affected relationship to mother					
Yes	0 (0)	1 (9)	0 (0)	1 (10)	2 (6)
No	9 (100)	10 (91)	5 9100)	9 (90)	33 (94)
Whether lack of genetic/gestational connection affected relationship to father					
Yes	0 (0)	0 (0)	0 (0)	0 (0)	0 (0)
No	9 (100)	11 (100)	5 (100)	10 (100)	35 (100)
Know others conceived in same way					
Yes	2 (22)	3 (27)	1 (20)	4 (40)	10 (29)
No	7 (78)	8 (73)	4 (80)	6 (60)	25 (71)
Member of donor conception or surrogacy group					
Yes in the past	1 (11)	0 (0)	0 (0)	0 (0)	1 (3)
No	8 (89)	11 (100)	5 (100)	10 (100)	34 (97)
Searched for information on conception					
Yes	5 (56)	6 (55)	4 (80)	1 (10)	16 (46)
No	4 (44)	5 (45)	1 (20)	9 (90)	19 (54)
Contact with donor or surrogate					
Yes	0 (0)	1 (9)	4 (80)	5 (50)	10 (29)
No	9 (100)	10 (91)	1 (20)	5 (50)	25 (71)

### Change in feelings about conception over time

Whilst the majority of young adults reported always feeling the same about their conception, for some (10/35) their feelings had changed over time, all but one of whom (9/10) had become more positive. This appeared to coincide with having a better understanding of what the treatment involved, for example:

*‘When I was first told, I didn’t really care, probably young enough to not care kind of thing, then as I got into the teenage years, uh, it somewhat, I’d kind of forgotten, and it kind of dawned on me that actually, this isn’t normal, but it didn’t bother me too much. I think it started to bother me more as a teenager, but then nowadays, I think I quite like it, it's different’.* Young adult born through egg donation

Similarly, another young adult commented on their feelings changing as they understood more about what their parents had gone through:

*‘Maybe in some ways I’ve become more aware, a bit more sympathetic to like the struggle my parents went through …’* Young adult born through surrogacy

One young adult acknowledged that he may feel differently in the future, particularly in relation to his interest in the donor:

‘*Erm … I think they’ve [my feelings] always been the same, I’ve never been too bothered about it, always just thought it was very cool and listening to what my dad and mum had to say was an interesting story. But I don’t know, I’m getting older now so I’ll be interested to see how I feel in the next few years …’* Young adult born through egg donation

### Frequency and ease of discussions

The participants rarely discussed their conception with others, with 22 young adults (63%) reporting that their discussions about their conception were infrequent or rare (Table II). For example, one young adult commented:

*‘… it’s nothing I really dwell on. I usually have more important things to really care about and as I said, it doesn’t affect anything’.* Young adult born through surrogacy

Another young adult said:

*‘It’s not like I ever forget, like oh yeah, I’m a sperm donor baby, you know, because it’s always been knowledge that technically dad isn’t my biological father, so yeah, it’s never that I forget that I’m a sperm donor [baby], but it’s never really on my mind …’.* Young adult born through sperm donation

However, when these conversations did come up with family and friends, they were generally easy conversations to have.

*‘I don’t mind them really. It’s like a normal conversation when you’re trying to explain who you are to someone else. It’s rather easy to do, it doesn’t put me off. I don’t mind it all’*. Young adult born through surrogacy

Only one young adult felt that discussions about his conception were difficult.

*‘I find it a little bit awkward, but I don’t find it like really uncomfortable or anything, it’s just a little bit … a little bit strange. I guess partly cause it’s just a conversation that people don’t often have, you know, so it can be a little bit strange’.* Young adult born through sperm donation

None of the young adults felt that others responded negatively to their method of conception currently, with many reporting that others were interested or curious to learn more.

### Openness of communication

As can be seen in Table II, young adults discussed their conception most openly with their family members, mainly their mothers (14, 40%). None discussed their conception more openly with their father than their mother, although some (6, 17%) reported the same level of openness with each parent.

### Whether lack of genetic/gestational connection affected relationship to parents

Young adults were asked whether their relationship with their parents had been affected by the absence of a genetic or gestational relationship to them. The majority of young adults did not feel that their relationship with either their mother or their father had been affected (Table II). For example, one young adult born following egg donation said:

‘*No … we still fight like, we still bicker about anything, but it doesn’t matter, doesn’t change anything, not at all’.”*

However, two young adults reported that the lack of a genetic or gestational connection to their mother had a negative impact on their relationship with her, with one born through traditional surrogacy referring to the lack of similarity with their mother, and the other born following egg donation reporting:

*‘Yeah I’ve always felt like a bit distant from her [mother] really’.*

### Knowing others born through same conception method

The majority of young adults did not know others born using the same type of conception as themselves (7/9 sperm donation, 8/11 egg donation, 4/5 gestational surrogacy, and 6/10 traditional surrogacy) (Table II). Three of these young adults acknowledged that they may know someone without being aware of it, as they themselves did not always tell others about their birth through surrogacy or gamete donation. For example, one adult born using surrogacy said:

*‘Uh, I don’t, no, is the short and simple answer. I mean, if I did, I don’t think they’d tell me, I wouldn’t tell them, it's not a conversation that ever comes up*’.

For the 10 young adults who knew others born through the same conception method as themselves, this was either because they knew them through school or through their parents, or for young adults born following traditional surrogacy, because they knew other children their surrogate had carried.

### Searching for information related to method of conception

Just over half of the young adults (54%, 19/35) had not searched for any information about their donor conception and none was currently a member of a donor conception or surrogacy group, although one had attended a meeting in the past (Table II).

For the 16 young adults who had searched for information, their reasons for searching were varied from searching in order to complete a school project, searching for who the first surrogacy child was, or to understand current legislation. Only one young adult had put in a request to the HFEA for non-identifying donor information, with a further two having looked on the internet to find out whether they could request information about their donor, but not initiating the request.

### Contact with donor or surrogate

One (9%) young adult born through known egg donation, five (50%) young adults born through traditional surrogacy, and four (80%) young adults born through gestational surrogacy were in contact with their donor or surrogate. The closeness of the relationship varied from being friends on social media, where contact was not active, but they were able to see each other’s news and photos, to seeing their surrogate frequently in person and describing their relationships as close. For the 25 not in contact with their surrogate or donor, 32% (8) did not want to have contact (four sperm donation, two egg donation, one traditional surrogacy, and one gestational surrogacy) and 44% (11) said they wished to meet them (two sperm donation, six egg donation and three traditional surrogacy). The remaining six (24%) were unsure about wanting to meet the donor/surrogate.

For those wishing to meet their donor or surrogate, this was often because they were interested in finding out particular characteristics about them, for example, what they looked like, or why they donated, or to thank them.

## Discussion

Most young adults in this study felt unconcerned about having been born through gamete donation or surrogacy. Although their method of conception was rarely discussed, when it did come up these conversations were conducted with ease and were rarely difficult or awkward. These positive findings may be related to the age at which these young adults had been told about their conception, with the majority of the present sample having been told by age 4 years. It may also reflect characteristics of our sample, which unlike many other studies of donor conceived adults, did not rely on a convenience sample recruited through support groups. Our findings are similar to those of other studies of families where the child had been told from a young age. For example, the Scheib *et al.* (2005) study of adolescents born following identity release donation found that most felt comfortable about their parents’ use of a donor. Previous studies have also found, similar to the present study, which feelings about the method of conception may change over time and can fluctuate between positive and negative emotions (Harrigan *et al.*, 2015). The present study extends these findings to young adults born following anonymous donation, and also to those born through surrogacy.

The UK Longitudinal Study of Assisted Reproduction Families is the only investigation to have followed up children born through surrogacy, and the findings from the present phase are similar to earlier phases (Jadva *et al.*, 2012; Zadeh *et al.*, 2018), showing that individuals born through surrogacy are mostly unconcerned about the method of their conception. For the surrogacy born young adults who were in contact with their surrogate, the amount of contact varied, as did the closeness of the relationship, from some reporting close relationships to others being Facebook friends, with no active direct contact. Regardless of the extent of contact, all the young adults felt positive about their birth through surrogacy, and those who wanted to contact their surrogate wished to do so because they were curious, or wished to thank her, and not because they felt a need to form a relationship with her.

The present study found that young adults discussed their conception most openly with their family, especially with their mothers. Studies of adoptive families have found that open communication, where conversations between parents and their children are open, honest, and non-defensive, is associated with fewer identity problems (Stein and Hoopes, 1985), more trust in their parents (Kohler *et al.*, 2002) and more satisfaction with the adoption experience (Howe and Feast, 2000). Indeed, openness of communication has been found to be a better predictor of child adjustment than whether or not the adopted child had contact with their birth parents (Brodzinsky, 2006; Farr *et al.*, 2014). The present study found that most young adults felt content about their conception, and this was the case amongst those who were in contact with their donor or surrogate as well as those who were not. It is important to note that, given that the families had been taking part in this longitudinal study from when the target child was aged 1 year, these families may have been more likely to discuss the child’s conception than other families. Future studies should aim to examine communication styles within families and how this may relate to how people born following third-party reproduction feel about their birth.

Although 44% of young adults in the present study who were not in contact with their donor or surrogate said that they may wish to meet them in the future, only one participant had directly sought information about them. A recent survey of donor conceived individuals born following identity-release sperm donation in Sweden found that only 7% had requested information about their donor (Lampic *et al.*, 2022). In the USA, an investigation by Scheib *et al.* (2017) found that 23% of young adults conceived by identity-release sperm donation from heterosexual-couple households had requested this information. Although the number of individuals who were aware of their conception in these studies was not known, Scheib *et al.* (2017) estimated that 85% of the parents in their study may have informed the child.

A reason for the young people in the present study not actively searching for their gamete donor is likely to be that the donor was anonymous. Therefore, they would not have had any expectation of discovering their donor’s identity, or of meeting them. It may also be linked to low levels of curiosity, as participants did not express an urgent need to obtain donor information. In a study of 21- to 30-year-old adoptees, a strong association was found between intensity of curiosity and information seeking about birth parents (Wrobel *et al.*, 2013), which may also be true of young people who are donor conceived. It is possible that feelings about actively searching for donor information may change in the future (Macmillan *et al.*, 2021). Jadva *et al.* (2010) found that almost one-third of donor conceived adolescents and adults reported that their search for their donor was prompted by a change in personal circumstances, such as becoming an adult, planning to, or having, children, and forming a long-term relationship. The same has been found among adopted adults (Howe and Feast, 2000).

It is important to note that the study utilized self-report measures, which may have been prone to social desirability where donor conceived young adults may have wanted to present their experiences in a positive light. However, the in-depth interview was designed to minimize socially desirable responding, and many of the young people were open about negative feelings and experiences in other aspects of their lives. It is also not known whether the young adults who did not take part in this phase of the study would feel differently to those who did. A particular strength of this study is in the representativeness of the original sample, as the donor-conception families were recruited systematically through clinics, and the surrogacy families were largely recruited through the Office of National Statistics. Moreover, the young adults had been followed up since infancy, and thus their recruitment to the study was not influenced by their feelings about the nature of their birth. None of the participants was a member of a support group related to their conception, and many had not actively searched for information about their origins. Thus, they are more representative of young people born through third-party assisted reproduction than those recruited thorough websites, social media, donor matching services, and support groups. The findings that this more representative sample of young adults born following third-party reproduction were largely unconcerned about their birth, were mostly able to openly discuss their conception with others, and were not actively searching for their donor or surrogate, fills an important gap in the literature.

The present study draws from a sample of donor conceived young adults with varied levels of information about the donor. Most of the young adults born following egg and sperm donation were born using an anonymous donor, and of those born following surrogacy, some were born using the egg of the surrogate and some young adults were in contact with their surrogate. Despite the variation in contact with the donor and surrogate, and in whether they were genetically related to their surrogate, the vast majority of young adults in the study shared largely similar feelings of being unconcerned about their conception. Some young adults who were not in contact with their donor or surrogate, were curious about them. This finding has important implications for considering whether donor information should be available ‘at use’, i.e. at the point of treatment. Having this information at the time of treatment would mean that parents could pass on this information to their children when their child requested it. However, this may not guarantee that donor conceived people will have access to this information, suggesting that the ability for donor conceived/or surrogacy born young adults to access this information later on, and independently of their parents, may be beneficial.

In conclusion, our findings show that young adults born through third-party reproduction feel positive or indifferent about their conception and their relationships with surrogates and known donors, where there is contact, appear to be good, although varied, in terms of strength and closeness. The majority of those born through anonymous gamete donation was not actively searching for their donor. With the first young adults born through identity release donation in the UK becoming eligible to request identifying information about their donor in 2023, it remains to be seen whether they will have different expectations from those with anonymous donors, and whether they will be more inclined to seek donor information.

## Data Availability

The data underlying this article cannot be shared publicly in order to maintain the privacy of individuals that participated in the study.
